# Crispr/Cas Mediated Deletion of PTPN22 in Jurkat T Cells Enhances TCR Signaling and Production of IL-2

**DOI:** 10.3389/fimmu.2018.02595

**Published:** 2018-11-12

**Authors:** Cara Bray, David Wright, Sonja Haupt, Sharyn Thomas, Hans Stauss, Rose Zamoyska

**Affiliations:** ^1^School of Biological Sciences, Institute for Immunology and Infection Research, University of Edinburgh, Edinburgh, United Kingdom; ^2^Institute of Immunity and Transplantation, Royal Free Hospital, University College London, London, United Kingdom

**Keywords:** PTPN22, CRISPR, Jurkat, T cell signaling, autoimmunity

## Abstract

A single nucleotide polymorphism, C1858T, in the gene encoding the protein tyrosine phosphatase nonreceptor type 22 (*PTPN22*) results in one of the strongest genetic traits associated with autoimmune disease outside of the Major Histocompatibility Complex (MHC) genes. However, the consequences of this polymorphism, which introduces an arginine to tryptophan substitution at amino acid 620, for the function of PTPN22 protein is unclear and conflicting results have been obtained in human compared to mouse cells expressing this variant phosphatase. In mouse the variant appears to be a loss-of-function allele resembling a milder form of the null allele, while studies in human cells have reported it to be a gain-of-function mutation. To address whether the phosphatase has distinct functions in mouse vs. human T cells, we used CRISPR gene-editing to generate the first example of human PTPN22-KnockOut (KO) T cells. By comparing isogenic human T cells which express or lack PTPN22, we showed that PTPN22 KO T cells displayed enhanced expression of IL-2 and CD69 upon stimulation with cognate antigen. PTPN22 KO cells also showed increased Erk phosphorylation upon stimulation with weak antigen, but the difference was diminished in response to strong antigen, indicating that PTPN22 plays a more critical role in regulating weak-antigen responses. These data are in keeping with a role for PTPN22 in determining the threshold of stimulation required to activate T cells, a critical function of autoimmune pathogenesis. Our data indicate that PTPN22 has comparable functions in mouse and human T cells, and that the conflicting results in the literature regarding the impact of the point mutation are not due to differences in the activity of PTPN22 itself, but may be related to interactions with other proteins or splice variation.

## Introduction

The protein tyrosine phosphatase nonreceptor type 22 (PTPN22) exhibits a common single nucleotide polymorphism (SNP), C1858T, which has been strongly linked with autoimmune disorders. Genome-wide association studies have connected the PTPN22^C1858T^ SNP with increased susceptibility to numerous autoimmune diseases, such as type 1 diabetes, rheumatoid arthritis, and systemic lupus erythematous, suggesting the variant allele plays a role in a common pathway in autoimmune development, however the mechanism behind the association is as yet unknown (1).

One aspect of the role of PTPN22 in autoimmune diseases may lie in its function in T cells. Mice deficient in PTPN22 exhibit enhanced T cell receptor responses, expanded effector/memory T cell populations, and enlarged germinal centers (2). PTPN22 acts as a negative regulator of T cell receptor signaling by dephosphorylating activating tyrosine residues on the proximal signaling molecules Lck, Fyn, and Zap-70 (3). PTPN22 has been shown to be particularly important in regulating responses to weak peptide stimulation in mouse T cells thus modulating self-reactivity without impairing responses to pathogens (4).

The autoimmune-associated SNP converts arginine 620 to a tryptophan (R620W) in a region of PTPN22 that mediates interaction with other proteins, and greatly reduces its association with another negative regulator of T cell signaling, C-terminal Src kinase (Csk). The functional significance of the loss of association between PTPN22 and Csk and its impact on autoimmune predisposition remains unresolved. Studies of the homologous mutation in mouse models have clearly demonstrated that the autoimmune-associated SNP is loss-of-function, leading to a reduced ability of PTPN22 to downregulate T cell responses: T cells isolated from these mice have enhanced activation profiles and cytokine production (5, 6), similar to observations in mice lacking PTPN22 altogether (2). However, many studies of human T cells expressing the PTPN22 R620W variant allele have yielded the opposite result: PTPN22 R620W appears to have a stronger negative regulatory effect, that is, it acts as a gain-of-function mutation compared to the more common form of the protein, resulting in reduced T cell effector functions (7, 8). On the other hand, PTPN22 R620W appeared to have a loss-of-function effect in the context of myasthenia gravis (9), and Zikherman et al demonstrated that overexpression of Csk as well as PTPN22 R620W revealed a loss-of-function effect for the SNP (10). Thus, the functional significance of the PTPN22^R620W^ mutation in human is not as straightforward as the clear loss-of-function effect seen in mice carrying the equivalent PTPN22 R619W alleles.

A possible source of the conflicting results in studies of human PTPN22 is the inability to compare the normal effects of the protein on a genetically identical background. Instead studies have either relied on material from genetically and environmentally distinct individuals, on overexpression of PTPN22 in cell lines, which alters the normal stoichiometry of the protein, or on siRNA knockdown methods or chemical inhibitors, which carry concerns regarding possible off-target effects.

Here, we address the challenges in studying the function of human PTPN22 by using CRISPR to create isogenic human T cell lines with PTPN22 knocked out at the genomic level, enabling us to isolate the effects of human PTPN22 on T cell signaling and compare whether it has a similar function to that seen in mouse T cells. We found that the role of PTPN22 in human T cell regulation is indeed similar to that in mice, in that its absence leads to enhancement of T cell signaling markers, especially in the context of weak antigen stimulation. These findings suggest that the discrepancies reported in the literature regarding the function of mouse and human PTPN22^R620W^ are not a consequence of distinct functions of the common allelic variant of the protein.

## Materials and methods

### Cell lines

TCR^−/−^ Jurkat cells and Phoenix-AMPHO cells were obtained from Hans Stauss, University College London. Cells were maintained in IMDM (Sigma-Aldrich) media supplemented with 5% heat- inactivated FBS (Serotec), 2 × 10^−3^ M L-glutamine, 100 U/ml penicillin, 100 mg/ml streptomycin, and 5 × 10^−5^ M 2-mercaptoethanol (Sigma-Aldrich). Retroviral transduction of TCR constructs was performed as described (11).

### Antibodies

Antibodies used for T cell stimulation included DimerX I recombinant HLA-A2 Ig (BD Biosciences), CD3 (clone OKT3, Biolegend), and CD28 (clone CD28.2, Biolegend). Western blot antibodies included PTPN22 (clone D6D1H, Cell Signaling Technology), Lck (Cell Signaling Technology), Zap-70 (Cell Signaling Technology), α-tubulin (clone TU-02, Santa Cruz), anti-rabbit Ig (Life Technologies) and anti-mouse Ig (LI-COR). For IP-FCM, the Akt capture antibody used was clone SKB1 (Merck Millipore), total Akt was detected using clone 53G (Cell Signaling Technology), and phospho Akt S473 was detected using clone D9E (Cell Signaling). Flow cytometry antibodies included CD69 (clone FN50, BD Biosciences), Il-2 (clone MQ1-17H12, Biolegend), pSrc family Y416 (Cell Signaling Technology), Lck pY505 (Cell Signaling Technology), Zap-70 pY493 (Cell Signaling Technology), Erk 1/2 pY204 (clone 197G2, Cell Signaling Technology), NFAT (clone D43B1, Cell Signaling Technology), cFos (clone 2G2, Novus Biologicals), an anti-rabbit secondary (Invitrogen), and an anti-mouse secondary (Life Technologies).

### CRISPR transfection

Cas9 plasmids Px330 and Px461 were purchased from Addgene and guide sequences were annealed as described (12). Neon transfection of plasmids into cells was carried out as per manufacturer protocol for Jurkat cells (Thermo Fisher).

### T cell stimulation

Stimulating peptides used were the HLA-A2-binding peptides pTax (LLFGYPVYV) and pHuD (LGYGFVNYI). DimerX I HLA-A2 Ig (BD Biosciences) was incubated overnight with 160 molar excess of peptide. Plates were coated with 20 μg/ml of DimerX+peptide or anti-CD3ε antibody. 2 μg/mL anti-CD28 was included in media for stimulation.

### T cell signaling assays

Intracellular cytokine staining was performed using the BD Cytofix/Cytoperm kit. Samples for phosphoprotein flow cytometry were prepared according to BD Phosflow protocol before being stained with primary and secondary antibodies.

For qPCR, RNA was isolated by Trizol and isopropanol precipitation. 1 μg of RNA was used for each reverse transcription reaction. qPCR mix was prepared according to SYBR Green manufacturer protocol (Thermo Fisher).

### qPCR primers

IL-2: CTTGCATTGCACTAAGTCTTGC & TAAATGTGAGCATCCTGGTGAG;

cFos: CCGGGGATAGCCTCTCTTACT & CCAGGTCCGTGCAGAAGTC;

cJun: CCTTGAAAGCTCAGAACTCGGAG & TGCTGCGTTAGCATGAGTTGGC

EGR-1: CTTCAACCCTCAGGCGGACA & GGAAAAGCGGCCAGTATAGGT

Akt analysis by immunoprecipitation of protein complexes by flow cytometry (IP-FCM) (13), western blotting, and calcium flux protocols have been previously described (14).

### Nucleus flow cytometry

Samples were stained using the recently described protocol (15). Briefly, samples were kept on ice unless otherwise noted. Cell pellets were resuspended in sucrose buffer containing 320 mM sucrose, 10 mM HEPES pH 7.8, 8 mM MgCl_2_, 0.1% Triton-X 100, and 1x Protease Inhibitor (Sigma-Aldrich P8340) and incubated for 15 min. Samples were then centrifuged at 2,000 × g for 10 min at 4°C. Pellets washed twice in washed in sucrose buffer lacking Triton-X. Nuclei were fixed by resuspending in sucrose buffer lacking Triton-X and containing 4% paraformaldehyde, and incubating for 25 min at room temperature, protected from light. Fixed nuclei were washed once with FACS buffer supplemented with 8 mM MgCl_2_ and centrifuged at 1,000 × g for 5 min at 4°C, then washed again and stained with flow cytometry antibodies diluted in PBS containing 2% FCS, 8 mM MgCl_2_, and 0.3% Triton-X 100. After staining, samples were washed and resuspended in FACS buffer containing MgCl_2_ for flow analysis.

## Results

### Generation of PTPN22 KO jurkat clones

We described previously that the impact of loss of PTPN22 in mouse OT-1 T cells varied depending on the strength of the stimulating pMHC ligand (4). In order to create a human T cell model which could be stimulated with strong and weak affinity peptides, we transduced TCR^−/−^ Jurkat cells with retrovirus encoding a TCR that has strong affinity for a pTax peptide (LLFGYPVYV, derived human T-lymphotropic virus type 1) and weak affinity to the pHuD peptide (LGYGFVNYI, derived from human neuronal protein), both of which are HLA-A2 restricted (11). Transduced cells were sorted for expression of Tax TCR and single cells cloned by limiting dilution. Clonal populations were expanded and tested for upregulation of activation markers when stimulated with pTax peptide. One clone with high expression of Tax TCR and reliable responsiveness to pTax was selected to serve as the parent line for CRISPR experiments.

**Figure 1:**
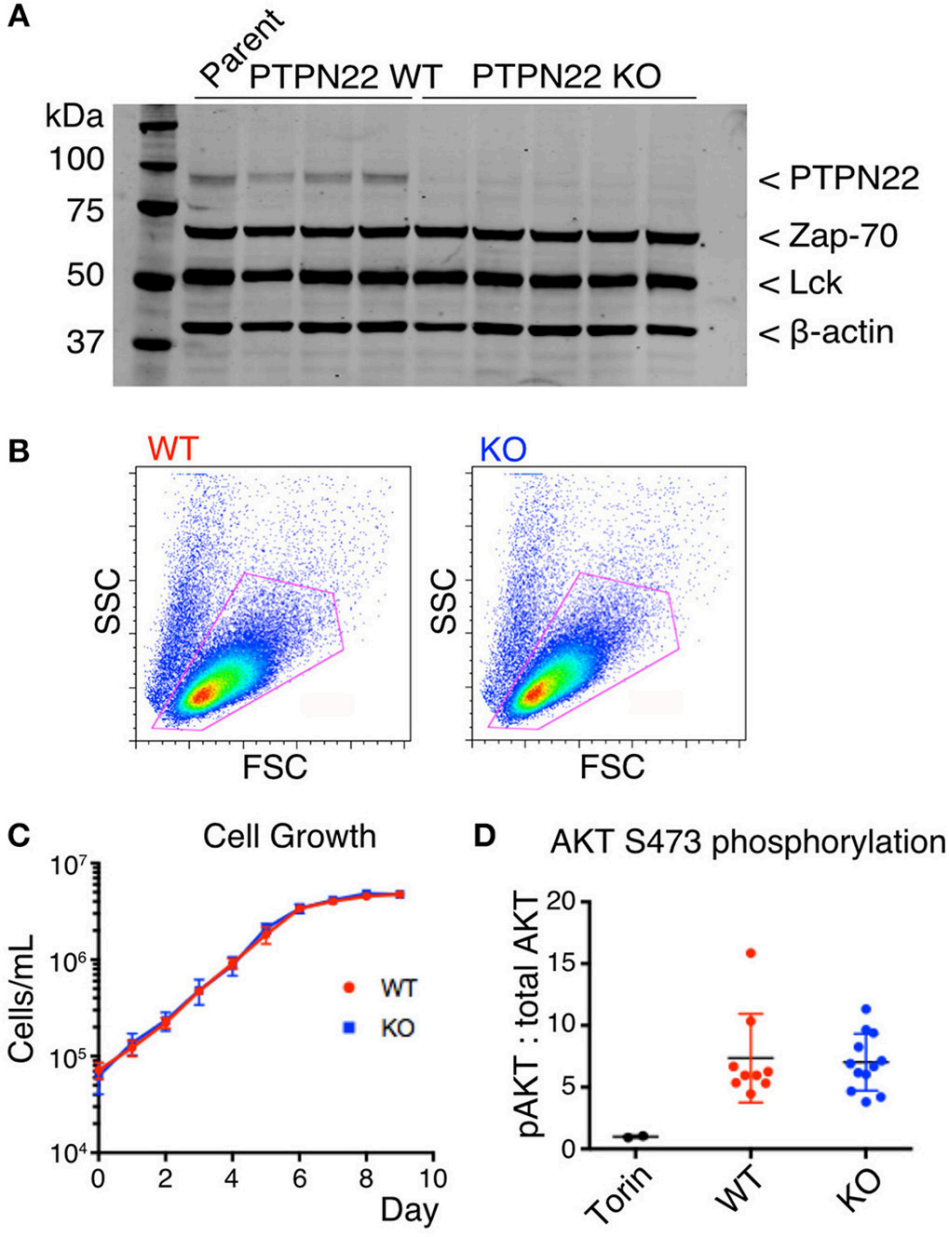
PTPN22 does not affect steady-state survival of Jurkat cells. **(A)** Expression of PTPN22, Zap-70, and Lck in 3 PTPN22 WT and 6 independently derived KO Jurkat clones were evaluated by Western blot, probed sequentially with Abs to PTPN22, Zap70, Lck, and β-actin as loading control. **(B)** No differences in cell size or granularity were observed between PTPN22 WT and KO Jurkat clones by flow cytometry in a representative clone of each. **(C)** PTPN22 WT and KO Jurkat clones were grown in culture and the number of cells was counted daily. Data is representative of two independent experiments. **(D)** Constitutive phosphorylation of AKT was evaluated by IP-FCM. The MFI of pAKT was normalized to the MFI of total AKT for each IP. Data from two independent experiments is shown.

The selected Tax TCR Jurkat clone was used for transfection with plasmids containing Cas9, sgRNA sequences targeting PTPN22 Exon 1, and a GFP reporter. A transfection was also performed on the same cells using only a GFP reporter construct to generate PTPN22 wild-type controls which have undergone the same manipulation (sgRNA sequences listed in Supplementary Figure 1). In both cases, GFP positive cells were sorted singly into wells and clonal populations were expanded. An initial screen was performed by PCR of the PTPN22 exon 1 region, and clones with visible shifts in the size of the PCR product were selected for further screening by western blot. An indel frequency of 70% was estimated based on the initial PCR screens. Western blot revealed that PTPN22 protein expression was completely lost in approximately half of the selected the clones, and we found that loss of PTPN22 expression did not influence expression of its known interactors Lck or Zap-70 (Figure 1A). The PTPN22 Exon 1 region of the clones was sequenced to confirm the deletion and similar expression of Tax TCR to the parent line was confirmed for each clone (Supplementary Figure 2). Six PTPN22 WT and six KO clones were selected for further studies.

### PTPN22 does not affect steady-state survival of jurkat cells

PTPN22 wild-type (WT) and knockout (KO) clones were identical in size and granularity as assessed by FACS analysis of forward and side scatter (Figure 1B). We detected no differences in growth of cultures between PTPN22 WT and KO Jurkat clones (Figure 1C), in contrast to the report by Baghbani et al., who found that siRNA disrupting PTPN22 transcription caused apoptosis in Jurkat cells (16). Baghbani et al. attributed their observations to reduction in phosphorylation of Akt in cells treated with PTPN22 siRNA, however we observed no alterations in Akt phosphorylation between PTPN22 WT and KO clones as assessed by IP-FACS (Figure 1D). As the maintenance of Jurkat cells in culture is a TCR-independent process it is perhaps not surprising that PTPN22 is not relevant in this context.

### Increased IL-2 and CD69 expression in PTPN22 KO jurkat cells

**Figure 2:**
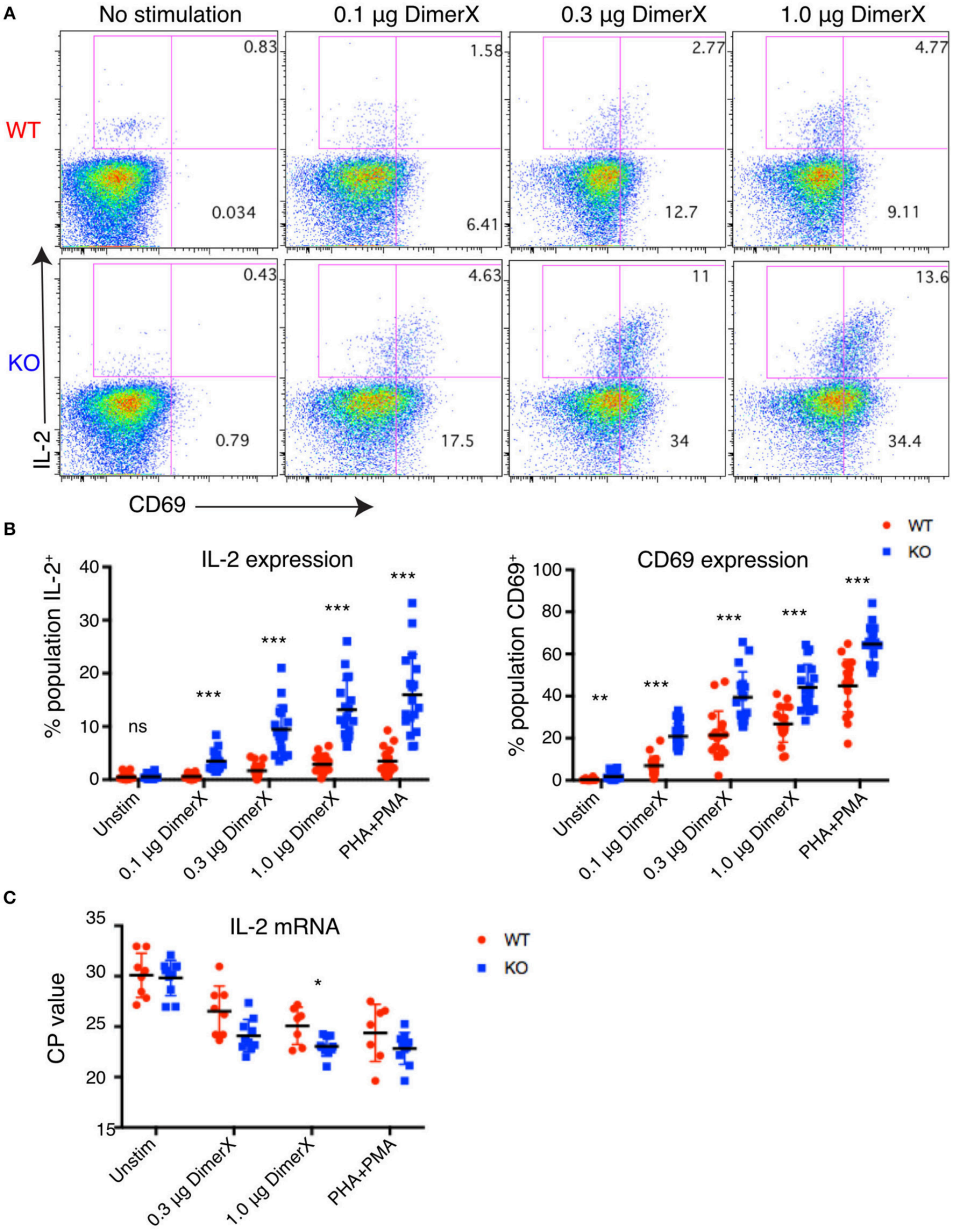
Increased IL-2 and CD69 expression in PTPN22 KO Jurkat cells. **(A)** Example dot plots of intracellular staining of representative Jurkat clones after 6 h DimerX+pTax stimulation. **(B)** Upregulation of IL-2 and CD69 in Jurkat clones was evaluated by intracellular staining and flow cytometry after 6 h of DimerX+pTax stimulation. Data represents three independent experiments which each included six clones of each genotype (*n* = 6). Each point represents a single clone in an experiment. **(C)** IL-2 mRNA was measured by qPCR after 4 h incubation with the indicated stimuli. Data represents three independent experiments which included three clones of each genotype (n = 3). Statistical significance for each condition was determined by unpaired *T*-test and Bonferroni multiple comparisons correction. A *p* < 0.05 was considered significant. ns, not significant, ^*^p < 0.05, ^**^p < 0.01, ^***^p < 0.001.

To determine the effect of PTPN22 on Jurkat T cell responses, we evaluated the upregulation of IL-2 and CD69 in response to cognate peptide. We loaded DimerX, a fusion protein linking human HLA-A2 to the variable heavy region of mouse IgG1, with pTax peptide and used this reagent bound to plastic 96-well plates to provide peptide-MHC stimulation of the clones. IL-2 and CD69 expression were evaluated by intracellular flow cytometry after 6 h of stimulation with DimerX + pTax in media containing anti-CD28 and Brefeldin A (Figure 2A).

We observed dose dependent upregulation of both IL-2 and CD69 after 6 h of stimulation and found that both were significantly higher in PTPN22 KO clones compared with PTPN22 WT clones. Increased responsiveness of PTPN22 KO clones was found at all concentrations of the antigen and with the PHA+PMA positive control stimulus (Figure 2B). PTPN22 KO clones also showed marginally elevated CD69 expression in the absence of stimulation, but this was much smaller than was observed following stimulation. The increase in expression of T cell activation markers in PTPN22 KO clones is similar to observations made in Ptpn22^−/−^ mice (2, 4, 17) and confirms that PTPN22 acts as a negative regulator in human T cells comparably to its role to mouse T cells.

To determine whether the increase in IL-2 production was driven by increased transcription, we measured the amount of IL-2 message by qPCR (Figure 2C). PTPN22 WT and KO clones were stimulated for 4 h with DimerX + pTax in media containing anti-CD28. We found that PTPN22 KO clones had significantly greater levels of IL-2 message (reduced *CP*-values), indicating that the increase in IL-2 expression can be attributed to increased transcription rather than any post-transcriptional regulation of mRNA.

### Altered responsiveness of PTPN22 KO cells is not detectable in early TCR signaling pathways

**Figure 3:**
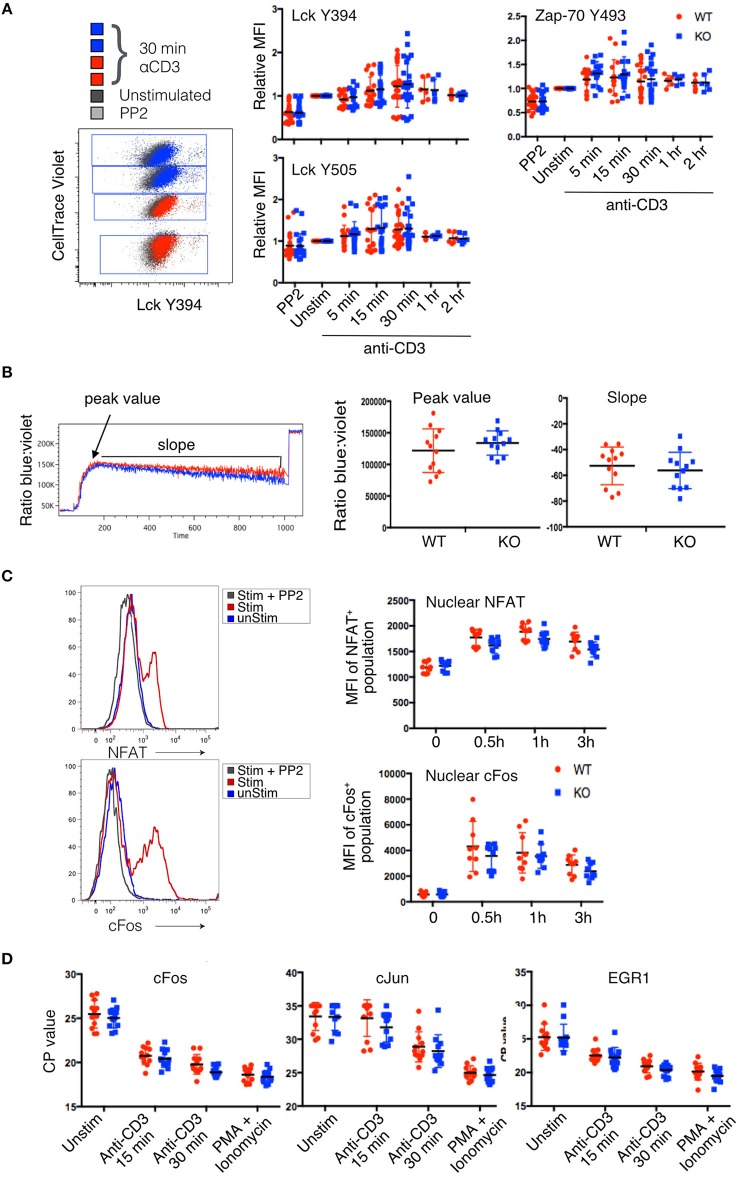
Lack of detectable changes in proximal signaling pathways in PTPN22 KO Jurkat cells. **(A)** Phosphorylation of Lck and Zap-70 tyrosine residues was evaluated by phosphoprotein flow cytometry after stimulation with αCD3 for the indicated time periods. CellTrace Violet tagging of PTPN22 WT and KO clones stimulated and stained in the same well is shown as a representative dot plot. Pooled data from three independent experiments is shown. **(B)** Calcium flux was evaluated by Indo-1 staining. Peak value, peak time, and slope of calcium flux upon stimulation with αCD3 were quantified and compared between PTPN22 WT and KO clones. Data from two independent experiments is shown. **(C)** Translocation of transcription factors was evaluated using nuclear flow cytometry after DimerX+pTax stimulation for the indicated time periods. Data from three independent experiments is shown. **(D)** Transcription of immediate early genes was evaluated by qPCR after CD3 stimulation for the indicated time periods. Data from four independent experiments is shown.

Given that IL-2 transcription was upregulated in PTPN22 KO cells we sought to determine which specific TCR signaling pathways in the Jurkat clones might be enhanced by loss of PTPN22. The early signaling molecules Lck, Fyn and Zap-70 are known targets for PTPN22 phosphatase activity (3). We used phosphoprotein flow cytometry to check for differences in Lck and Zap-70 phosphorylation. DimerX HLA-A2 + pTax stimulation did not produce sufficient changes in phosphorylation of Lck and ZAP-70 to be detectable by FACS, possibly because this stimulus was be too weak or transient. Instead we used plate-bound anti-CD3 to stimulate the clones which does produce changes in phosphorylation of Lck and ZAP-70 which are detectable by FACS. In order to control for well to well variation in stimulation, two independently derived PTPN22 WT and two PTPN22 KO clones were differentially labeled with CellTrace Violet (CTV) and stimulated together in each well, so that the four clones were treated with identical stimulation and staining conditions (Figure 3A). Which individual clones were labeled with the different dye concentration was varied randomly between experiments to exclude any effects of the dye on the response or the detection of phosphorylated substrates.

A representative example of the staining from a single well is shown in Figure 3A. Although there was clear shift in the staining of stimulated clones (red and blue dots) compared to unstimulated (black dots) or inhibited (PP2-treated, gray dots) clones, the variance in the responses of individual clones in different experiments overall was too high to see any statistically significant changes between PTPN22 WT and KO cells. However, there was a very slight but consistent increase in the average phosphorylation of residues in stimulated PTPN22 KO cells, although this did not reach statistical significance. It is possible that the assay was not sensitive enough to report subtle differences in early transient phosphorylation events. These results are in keeping with our previous data (18) with primary mouse T cells which, following stimulation, showed only small changes in phosphorylation of these early kinases between WT and PTPN22 KO T cells with considerably less variance between samples than observed with these Jurkat clones. However, while the slight differences in early signaling molecule phosphorylation were not statistically significant, they might still have biological relevance and to be responsible for the changes in IL-2 production which are further downstream.

Calcium signaling was reported to be enhanced in PTPN22^−/−^ mouse T cells (10, 14, 19) and to be diminished in Jurkat cells overexpressing PTPN22 (8). To compare calcium signaling in PTPN22 WT and KO clones, we differentially labeled one clone of each genotype with CFSE, loaded both with Indo-1, and analyzed calcium flux in response to saturating amounts of aqueous anti-CD3 stimulation. We analyzed three parameters: peak value, the maximum ratio of violet to blue Indo-1 reached by a clone; peak time, the number of seconds until a clone reached its peak value; and slope, the rate at which the ratio of violet to blue Indo-1 returned to baseline. All three parameters were equivalent for each individual clone and data for the peak value and slope are shown in Figure 3B. Fifteen minutes after the addition of anti-CD3, cells were treated with ionomycin to observe the maximum flux per clone.

Although there was variation in the Ca^2+^ flux between individual clones stimulated together in the same well, this appeared to be clone specific and unrelated to the presence or absence of PTPN22. Overall no significant differences in calcium flux were detected when the data was pooled for multiple PTPN22 WT and KO clones tested for any of the parameters analyzed (Figure 3B). Once again there was a slight trend for PTPN22 KO clones to cluster toward the higher range of peak calcium flux value, which likely accounts for the slightly steeper slope as they returned to baseline, but overall the variance was such that this difference was not statistically significant.

Many pathways of TCR signaling culminate in the activation of the transcription factors NFAT, and AP-1, and their translocation into the nucleus, which is a key event for cytokine production. To determine whether enhanced translocation of these transcription factors occurred in PTPN22 KO vs. WT clones, we isolated nuclei from stimulated cells and analyzed them for the presence of NFAT1 and cFos by flow cytometry (15). We observed clear translocation of NFAT and cFos into the nucleus following stimulation which was inhibited by pre-treatment of the cells with PP2 indicating the translocation was linked to activation (Figure 3C). Surprisingly we did not see any difference between PTPN22 WT and KO clones over the time course of stimulation that was analyzed. It was puzzling that the increased frequency of IL-2 producing cells amongst the PTPN22 KO clones (Figure 2C) did not correlate with an increased nuclear import of key transcription factors known to be important for IL-2 transcription, however, presence of a transcription factor in the nucleus does not guarantee increased transcriptional activity of a particular genetic locus and furthermore only one member of each transcription factor family was analysed in this set of experiments.

To further investigate transcriptional activity triggered by TCR signaling, we used qPCR to compare the transcript abundance of immediate early genes, cJun, cFos and EGR1 in PTPN22 WT and KO clones. In this assay, a lower value for the crossing point (CP) cycle indicates more mRNA in the sample. Instead of normalizing values to a housekeeping gene, the abundance of which we found to fluctuate upon stimulation (Supplementary Figure 3), we normalized the loading of each cDNA reaction to an input concentration of 1 μg RNA. Again, no significant differences in the transcription of immediate early genes were detected between PTPN22 WT and KO clones, although a very slight trend was observed toward PTPN22 KO clones having lower *CP*-values and thus more mRNA (Figure 3D).

### PTPN22 has a greater regulatory effect on T cell responses to weak stimulus

**Figure 4:**
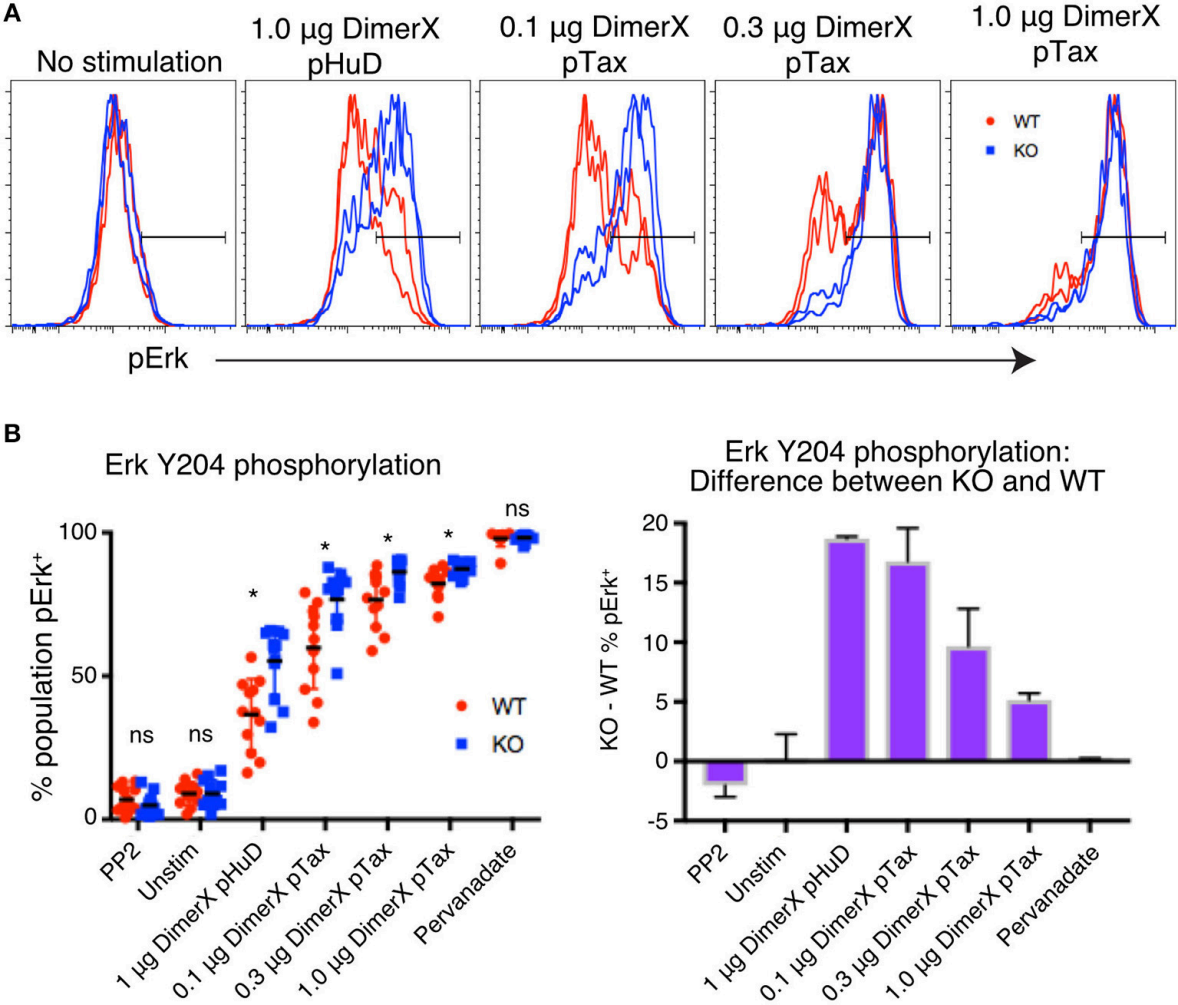
PTPN22 dampens ERK phosphorylation particularly in response to weak stimuli. **(A)** Representative histograms of phosphorylation of Erk Y204 in Jurkat clones, evaluated by phosphoprotein flow cytometry following 1 h of stimulation with the indicated amount of DimerX + peptide. Clones were tagged with CellTrace Violet and stimulated and stained in the same well. **(B)** Data from two independent experiments is shown. Statistical significance for each condition was determined by unpaired *T*-test and Bonferroni multiple comparisons correction. A *p* < 0.05 was considered significant. ns, not significant, ^*^p < 0.05.

Previous reports have suggested that PTPN22 may be more critical in regulating T cell responses to low-affinity stimulus in mouse (4). To test whether this function of PTPN22 is conserved in human T cells, we assayed Erk phosphorylation. pErk was consistently detected in over 90% of stimulated Jurkat cells and continued to be clearly detectable even after very weak stimulation, making it an ideal readout to study low-affinity peptide stimulation. As before, for consistency of stimulation, individual clones were labeled with varying concentrations of CellTrace Violet so that they could be activated and stained in the same well. 1-h stimulations were performed using titrations of plate-bound DimerX containing either pTax or the weak affinity peptide pHuD and pErk was measured by phosphoprotein flow cytometry (Figure 4A).

We found that frequency of cells expressing pErk was increased significantly in PTPN22 KO clones compared with WT clones, particulary in conditions of weak stimulation such as with the low affinity peptide pHuD or with low concentrations of the higher affinity pTax peptide. However, the difference between PTPN22 WT and KO responses was lost as stimulation strength increased (Figure 4B), demonstrating that PTPN22 KO clones specifically showed enhanced responses to weak stimuli, compared to PTPN22 WT clones. As we showed with mouse CD8 T cells, these results indicate that human PTPN22 is more significant in regulating T cell responses to weak peptide stimulation, but that its regulation is overridden as signal strength increases. Stimulation by weak antigen are critical drivers of autoimmune and cancer responses, thus this function of PTPN22 may be highly relevant to the development of these diseases.

## Discussion

We demonstrate here that the absence of PTPN22 in Jurkat cells leads to enhancement of certain T cell responses. Our findings showed that IL-2 and CD69 upregulation in response to stimulation was significantly enhanced in human T cells lacking PTPN22, which was similarly observed in mouse T cells (4, 14, 17, 20), suggesting that PTPN22 downregulates similar pathways of TCR signaling in human and mouse cells. Our data corroborated those from Perri et al. (21), who reported increased IL-2 expression in human T cells treated with PTPN22-siRNA. The evidence therefore supports a negative regulatory role for human PTPN22 of the pathways leading to IL-2 induction in T cells.

Given the clear increase in IL-2 producing cells amongst the PTPN22 KO clones, it was surprising that we could detect little evidence of differences in the upstream signaling pathways or activation of transcription factors leading to IL-2 production. However, the assays used to monitor signaling are, by and large, fairly insensitive. Anti-CD3 stimulation or saturating amounts of DimerX were often needed to acquire a signal above the detection threshold particularly in assays requiring short term stimulations including phosphorylation of proximal substrates such as Lck and ZAP-70 or Ca^2+^ flux. These concentrations of stimuli are on the upper plateau of the response where differences in the responses between two populations may be masked. There was also a wide variance of response in many of these assays between individual clones on different days, despite our attempts to minimize this variance by stimulating multiple individually labeled clones in a single well where possible. In contrast, we were able to detect differences between PTPN22 WT and KO clones using assays which report more downstream events such as ERK phosphorylation, IL-2 production and CD69 upregulation, indicating that the presence of PTPN22 in WT cells serves to modulate these responses and this modulation was most apparent following weaker stimulation.

We reported previously that weak stimulation led to stronger responses in PTPN22 knockout mouse OT-1 T cells, and the influence of loss of PTPN22 was reduced with increased stimulation strength (4). Our data here suggests that human PTPN22 has a greater regulatory effect on T cell signaling in contexts of weak peptide stimulation also. Thus, in human and in mouse, PTPN22 serves to differentiate weak TCR stimulation from strong stimulation, and specifically downregulates T cell signaling in the former situation. Whether there is a specific mechanism regulating PTPN22 activity according to stimulation strength or whether PTPN22 simply provides relatively weak negative regulation which is overruled by strong stimulus remains to be seen.

Based on our findings, the reported discrepancies between PTPN22 R620W studies in humans and mice, in which the former was reported to be a gain-of-function mutation (7, 8) while the latter showed a loss-of-function (5, 6) are unlikely to be due differences in the normal activity of full-length PTPN22, but perhaps instead to its interactions with other regulatory proteins that have yet to be fully described. Another possible root of the controversy between the conflicting mouse and human PTPN22^R620W^ data may be related to the reported presence of different isoforms of PTPN22 expressed by human T cells (22, 23). In contrast our Jurkat clones and mouse T cells appear to express only the full-length protein as determined by western blot. The roles of the various isoforms of human PTPN22 are only partially elucidated (22, 23), and may have different effects on the function of PTPN22 in individuals expressing the R620W variant allele. For example, Chang et al described a dominant negative isoform of human PTPN22, overexpression of which increased production of IL-2 in Jurkat cells (24). Additionally, differences in PTPN22 isoform ratios have been reported in patients with rheumatoid arthritis and systemic lupus erythematosus (25, 26). It is possible that these isoforms lend functions to human PTPN22 that are absent in mouse and Jurkat T cells, and which may be differentially affected by the R620W polymorphism.

The ability to knockout signaling molecules in human T cells using Crispr/Cas provides an unprecedented opportunity to compare the function of such molecules between mouse and human T cells. In the case of PTPN22, the data from the analysis of knockout cells reported here suggest this phosphatase has a similar function in both species to modulate T cell signaling.

## Author contributions

CB performed experiments and wrote the manuscript, DW performed experiments, SH contributed to experiments and writing of the manuscript, ST performed experiments, HS provided intellectual input and reagents, RZ conceived and supervised the work and wrote the manuscript.

### Conflict of interest statement

The authors declare that the research was conducted in the absence of any commercial or financial relationships that could be construed as a potential conflict of interest.

## References

[1] Gregersen PK Lee H-S Batliwalla F Begovich AB. PTPN22: setting thresholds for autoimmunity. Semin Immunol. (2006) 18:214–23. 10.1016/j.smim.2006.03.009 16731003

[2] Hasegawa K Martin F Huang G Tumas D Diehl L Chan AC. PEST domain-enriched tyrosine phosphatase (PEP) regulation of effector/memory T cells. Science (2004) 303:685–9. 10.1126/science.1092138 14752163

[3] Cloutier JF Veillette A. Association of inhibitory tyrosine protein kinase p50csk with protein tyrosine phosphatase PEP in T cells and other hemopoietic cells. EMBO J. (1996) 15:4909–18. 10.1002/j.1460-2075.1996.tb00871.x 8890164 PMC452228

[4] Salmond RJ Brownlie RJ Morrison VL Zamoyska R. The tyrosine phosphatase PTPN22 discriminates weak self peptides from strong agonist TCR signals. Nat Immunol. (2014) 15:875–83. 10.1038/ni.2958 25108421 PMC4148831

[5] Zhang J Zahir N Jiang Q Miliotis H Heyraud S Meng X. The autoimmune disease-associated PTPN22 variant promotes calpain-mediated Lyp/Pep degradation associated with lymphocyte and dendritic cell hyperresponsiveness. Nat Genet. (2011) 43:902–7. 10.1038/ng.904 21841778

[6] Dai X James RG Habib T Singh S Jackson S Khim S. A disease-associated PTPN22 variant promotes systemic autoimmunity in murine models. J Clin Invest. (2013) 123:2024–36. 10.1172/JCI66963 23619366 PMC3638909

[7] Rieck M Arechiga A Onengut-Gumuscu S Greenbaum C Concannon P Buckner JH. Genetic variation in PTPN22 corresponds to altered function of T and B lymphocytes. J Immunol. (2007) 179:4704–10. 10.4049/jimmunol.179.7.4704 17878369

[8] Vang T Congia M Macis MD Musumeci L Orrú V Zavattari P. Autoimmune-associated lymphoid tyrosine phosphatase is a gain-of-function variant. Nat Genet (2005) 37:1317–1319. 10.1038/ng1673 16273109

[9] Lefvert AK Zhao Y Ramanujam R Yu S Pirskanen R Hammarström L. PTPN22 R620W promotes production of anti-AChR autoantibodies and IL-2 in myasthenia gravis. J Neuroimmunol (2008) 197:110–13. 10.1016/j.jneuroim.2008.04.004 18533277

[10] Zikherman J Hermiston M Steiner D Hasegawa K Chan A Weiss A. PTPN22 deficiency cooperates with the CD45 E613R allele to break tolerance on a non-autoimmune background. J Immunol. (2009) 182:4093–106. 10.4049/jimmunol.0803317 19299707 PMC2765978

[11] Thomas S Xue SA Bangham CRM Jakobsen BK Morris EC Stauss HJ. Human T cells expressing affinity-matured TCR display accelerated responses but fail to recognize low density of MHC-peptide antigen. Blood (2011) 118:319–29. 10.1182/blood-2010-12-326736 21606483

[12] Ran FA Hsu PD Wright J Agarwala V Scott DA Zhang F. Genome engineering using the CRISPR-Cas9 system. Nat Protoc. (2013) 8:2281–308. 10.1038/nprot.2013.143 24157548 PMC3969860

[13] Schrum AG Gil D Dopfer EP Wiest DL Turka LA Schamel WWA. High-sensitivity detection and quantitative analysis of native protein-protein interactions and multiprotein complexes by flow cytometry. Sci STKE (2007) 2007:pl2. 10.1126/stke.3892007pl2 17551170 PMC3913565

[14] Sood S Brownlie RJ Garcia C Cowan G Salmond RJ Sakaguchi S. Loss of the protein tyrosine phosphatase PTPN22 reduces mannan-induced autoimmune arthritis in SKG Mice. J Immunol. (2016) 197:429–40. 10.4049/jimmunol.1502656 27288531 PMC4932175

[15] Gallagher MP Conley JM Berg LJ. Peptide antigen concentration modulates digital NFAT1 activation in primary mouse naïve CD8^+^ T cells as measured by flow cytometry of isolated cell nuclei. Immunohorizons (2018) 2:208–15. 10.4049/immunohorizons.1800032 30221251 PMC6135534

[16] Baghbani E Baradaran B Pak F Mohammadnejad L Shanehbandi D Mansoori B. Suppression of protein tyrosine phosphatase PTPN22 gene induces apoptosis in T-cell leukemia cell line (Jurkat) through the AKT and ERK pathways. Biomed Pharmacother. (2017) 86:41–7. 10.1016/j.biopha.2016.11.124 27936393

[17] Zheng P Kissler S PTPN22 silencing in the NOD model indicates the type 1 diabetes-associated allele is not a loss-of-function variant. Diabetes (2013) 62:896–904. 10.2337/db12-0929 23193190 PMC3581188

[18] Brownlie RJ Miosge LA Vassilakos D Svensson LM Cope A Zamoyska R. Lack of the phosphatase PTPN22 increases adhesion of murine regulatory T cells to improve their immunosuppressive function. Sci Signal. (2012) 5:ra87. 10.1126/scisignal.2003365 23193160 PMC5836999

[19] Maine CJ Hamilton-Williams EE Cheung J Stanford SM Bottini N Wicker LS. PTPN22 alters the development of regulatory T cells in the thymus. J Immunol. (2012) 188:5267–75. 10.4049/jimmunol.1200150 22539785 PMC3358490

[20] Brownlie RJ Garcia C Ravasz M Zehn D Salmond RJ Zamoyska R. Resistance to TGFβ suppression and improved anti-tumor responses in CD8^+^ T cells lacking PTPN22. Nat Commun. (2017) 8:1–10. 10.1038/s41467-017-01427-1 29116089 PMC5676842

[21] Perri V Pellegrino M Ceccacci F Scipioni A Petrini S Gianchecchi E. Use of short interfering RNA delivered by cationic liposomes to enable efficient down-regulation of PTPN22 gene in human T lymphocytes. PLoS ONE (2017) 12:e0175784. 10.1371/journal.pone.0175784 28437437 PMC5402975

[22] Cohen S Dadi H Shaoul E Sharfe N Roifman CM. Cloning and characterization of a lymphoid-specific, inducible human protein tyrosine phosphatase, Lyp. Blood (1999) 93:2013–2024. 10068674

[23] Wang S Dong H Han J Ho WT Fu X Zhao ZJ. Identification of a variant form of tyrosine phosphatase LYP. BMC Mol Biol. (2010) 11:78. 10.1186/1471-2199-11-78 21044313 PMC2987843

[24] Chang H-H Tai T-S Lu B Iannaccone C Cernadas M Weinblatt M. PTPN22.6, a dominant negative isoform of PTPN22 and potential biomarker of rheumatoid arthritis. PLoS ONE (2012) 7:e33067. 10.1371/journal.pone.0033067 22427951 PMC3299735

[25] Chang H-H Tseng W Cui J Costenbader K Ho I-C. Altered expression of protein tyrosine phosphatase, non-receptor type 22 isoforms in systemic lupus erythematosus. Arthritis Res Ther (2014) 16:R14. 10.1186/ar4440 24433447 PMC3979039

[26] Ronninger M Guo Y Shchetynsky K Hill A Khademi M Olsson T. The balance of expression of PTPN22 splice forms is significantly different in rheumatoid arthritis patients compared with controls. Genome Med. (2012) 4:2. 10.1186/gm301 22264340 PMC3334550

