# Supplementary material for: Crispr/Cas Mediated Deletion of PTPN22 in Jurkat T Cells Enhances TCR Signaling and Production of IL-2
Source: Front Immunol. 2018 Nov 12;9:2595. doi: 10.3389/fimmu.2018.02595 (PMC6240618; doi:10.3389/fimmu.2018.02595)
Supplement: Supplementary file 1 [file Data_Sheet_1.PDF]

A

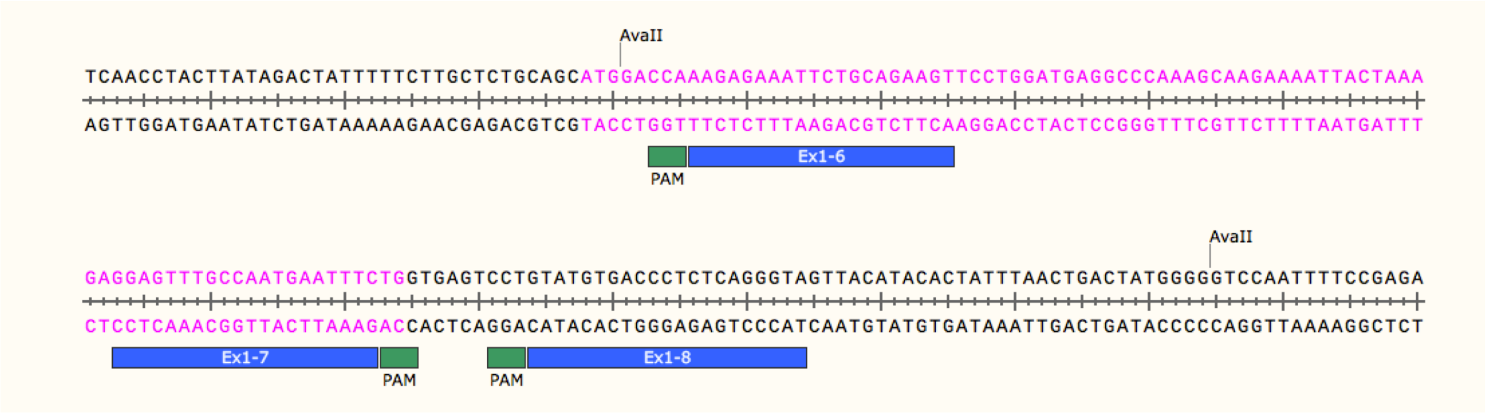

B

**sgRNA**		**Plasmid**
Ex1-6	ACTTCTGCAGAATTTCTCTT	Px330
Ex1-7	GGAGTTTGCCAATGAATTTC	Px461
Ex1-8	ACCCTGAGAGGGTCACATAC	Px461

Suppl Fig 1: CRISPR sgRNA sequences. (A) sgRNA sequence alignment with PTPN22 Exon 1 is shown. (B) sgRNA sequences and their associated Addgene plasmids are shown

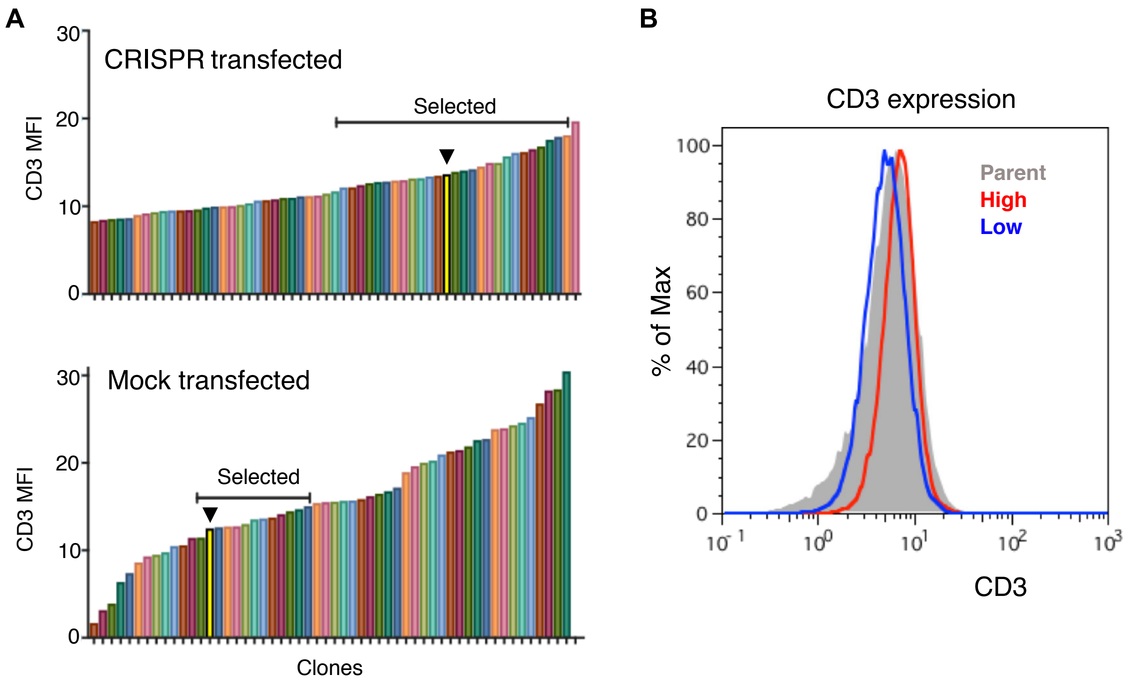

Suppl Fig 2. Selection of Jurkat clones by CD3 expression. (A) Clonal lines of Jurkat cells that had been transfected with CRISPR plasmids or with a GFP reporter plasmid were checked for CD3 expression by flow cytometry and compared to the parent line (yellow bar, indicated by arrow head) Clones with an MFI within 10% of the parent line were selected. (**b**) Histogram demonstrating the range of CD3 expression of selected clones. The C11 parent line is shown in grey, with the highest selected CRISPR clone in red and the lowest selected clone in blue.

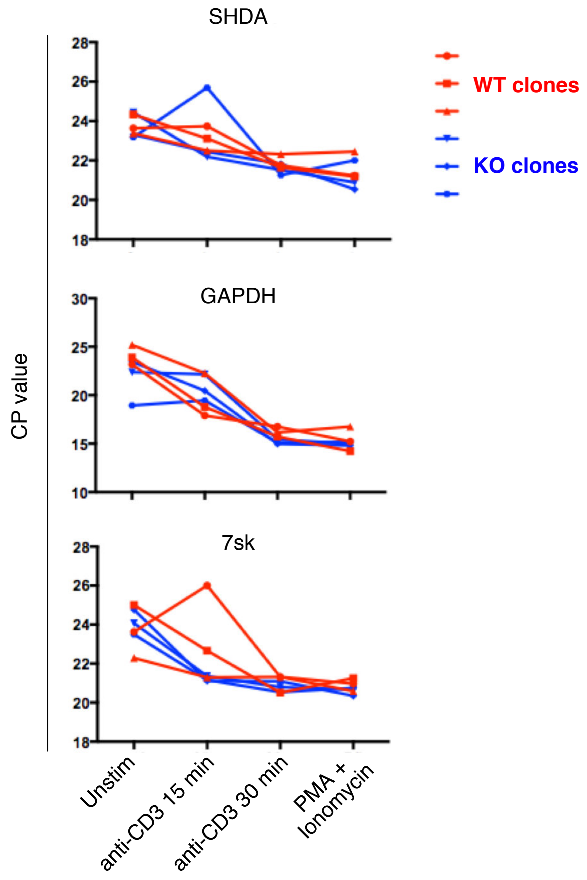

Suppl Fig 3: Housekeeping genes show increased mRNA expression upon stimulation. mRNA was isolated from PTPN22 WT and KO Jurkat clones after indicated treatments, and evaluated for expression of housekeeping gene transcripts. Data representative of 3 independent experiments and 3 clones of each genotype, WT and KO.
